# Curricular integration of social medicine: a prospective for medical educators

**DOI:** 10.3402/meo.v21.30586

**Published:** 2016-01-14

**Authors:** Allison A. Vanderbilt, Reginald F. Baugh, Patricia A. Hogue, Julie A. Brennan, Imran I. Ali

**Affiliations:** 1 Department of Family Medicine, College of Medicine and Life Sciences, University of Toledo, Toledo, OH, USA; 2 Department of Surgery, College of Medicine and Life Sciences, University of Toledo, Toledo, OH, USA; 3 Department of Physician Assistant Studies, College of Medicine and Life Sciences, University of Toledo, Toledo, OH, USA; 4 Family Medicine and Division, Adult Psychiatry, College of Medicine and Life Sciences, University of Toledo, Toledo, OH, USA; 5 Department of Neurology, College of Medicine and Life Sciences, University of Toledo, Toledo, OH, USA

**Keywords:** health disparities, social medicine, medical education, curriculum, LCME

## Abstract

In the United States, the health of a community falls on a continuum ranging from healthy to unhealthy and fluctuates based on several variables. Research policy and public health practice literature report substantial disparities in life expectancy, morbidity, risk factors, and quality of life, as well as persistence of these disparities among segments of the population. One such way to close this gap is to streamline medical education to better prepare our future physicians for our patients in underserved communities. Medical schools have the potential to close the gap when training future physicians by providing them with the principles of social medicine that can contribute to the reduction of health disparities. Curriculum reform and systematic formative assessment and evaluative measures can be developed to match social medicine and health disparities curricula for individual medical schools, thus assuring that future physicians are being properly prepared for residency and the workforce to decrease health inequities in the United States. We propose that curriculum reform includes an ongoing social medicine component for medical students. Continued exposure, practice, and education related to social medicine across medical school will enhance the awareness and knowledge for our students. This will result in better preparation for the zero mile stone residency set forth by the Accreditation Council of Graduate Medical Education and will eventually lead to the outcome of higher quality physicians in the United States to treat diverse populations.

The compilation of communities is the foundation of the overall health status of the population. Unfortunately, in the United States, the health of a community falls on a continuum ranging from healthy to unhealthy and fluctuates based on several variables. Alarmingly, these variables are linked to an enormous gap within the healthcare system related to health disparities that are commonly associated with a variety of factors, including insurance status, income, and race (1). Research policy and public health practice literature report substantial disparities in life expectancy, morbidity, risk factors, and quality of life, as well as persistence of these disparities among segments of the population (2–6). Health disparities can negatively impact subsets of the population who have systematically experienced greater social or economic obstacles to health. These obstacles can stem from characteristics historically linked to discrimination or exclusion such as race or ethnicity, religion, socioeconomic status, gender, mental health, sexual orientation, geographic location, cognitive, sensory, or physical disability (7).

It is essential that all healthcare providers work collaboratively toward the same common goal in systematically closing the health disparities gap within the United States. It is the duty of health-care professionals to collaborate together in order to reduce health disparities and better the overall health among people within the United States. One such way to close this gap is to streamline medical education to better prepare our future physicians for our patients in underserved communities.

This streamline effect can occur as early as medical school. Dopelt et al. (8) noted that medical education is a critical component of dealing with health disparities. In addition, medical schools have the potential to close the gap when training future physicians by providing them with the principles of social medicine that can contribute to the reduction of health disparities. Social medicine is the umbrella that encompasses health disparities, cultural competency, diversity, service, and population health. Therefore, a commitment to educating medical students and providing them with the foundation of social medicine will create physicians who are socially oriented and who are highly involved in activities that benefit the community (9). Some may argue that social medicine's focus on the intersection of medicine and society rather than medicine and the individual patient is misguided. Yet, the patient exists within a specific set of social conditions, within a specific context of medical care, possessing unique characteristics individually and as a member of more than one population. To best treat that individual, the context of care and knowledge of social medicine adds valuable tools and perspectives to optimize outcomes.

Medical schools have the potential to develop curriculum, standards, and criteria that can be implemented to reduce health disparities (10). Incorporating the principles of social medicine into medical education for trainings and/or curriculum is vital; it has been noted by several key professional agencies such as The Institute of Medicine, the Accreditation Council of Graduate Medical Education (ACGME), and the Liaison Committee on Medical Education (LCME) (11–13). This will allow for medical schools to measure their performance and commitment to reducing health disparities in the United States. Curriculum reform and systematic formative assessment and evaluative measures can be developed to match the social medicine health disparities curricula for individual medical schools with their mission related to assuring that future physicians are being properly prepared for residency and the workforce to decrease health inequities in the United States.

To achieve these target goals, medical school curriculum must be reformed to meet the needs of our incoming learners to best prepare them for the zero milestone residency, to provide quality care to patients, and to close the gap on health disparities within the United States. To better understand how to tackle closing the gap, medical educators must understand underserved population data and the requirements of the future physician workforce for critical need areas.

Disturbingly, 57 million individuals live in 5,864 designated primary care shortage areas in the United States (14). Individuals in these urban and rural communities face a paucity of primary care providers in four primary care specialties: general or family practice, general internal medicine, pediatrics, and obstetrics and gynecology (15). Primary care physicians compose only 37% of the physician workforce; however, they provide 56% of all physician office visits (16). Experts argue that the United States will face a critical shortage of primary care physicians in the near future (16), resulting in the reduction of access to primary care services for medically underserved individuals (17).

Researchers have found that medical students exposed to underserved populations during education and training are more likely to care for this same population once in practice (18); therefore, this may strengthen the health-care infrastructure in underserved communities (19). In fact, primary care physicians who complete residency training in community health centers (safety net providers for the uninsured and other vulnerable populations) are significantly more likely to practice in medically underserved areas (20). In addition, medical students who train with underserved populations are thought to learn and rediscover social responsibility and further understand the social determinants of health (21). Thus, it is essential for medical educators, faculty, and senior level leadership at medical schools to collaborate when working on curriculum reform to ensure that both the principles and practice of social medicine, especially as it applies to health disparities education, are included in curriculum reform.

When revising medical school curriculum, it is not sufficient to include a 1-hour lecture on health disparities to proclaim that medical students have been ‘taught’ about underserved populations and the LCME standards are now met. We propose that curriculum reform includes an ongoing social medicine component for medical students. Providing students with interactive awareness (22) experiences to become mindful of implicit bias; clinical rotations and electives focused on serving diverse communities and quality improvement projects that focuses on healthcare disparities throughout their years in medical school. This integrated pedagogic approach based on the literature (14–21) combining clinical experiences with didactic and self-reflection will have the biggest impact on undergraduate medical school learners and has the potential to increase the number of future physicians serving the populations of underserved areas. Furthermore, medical students will have the opportunity to participate in service learning as part of the community and interact with at-risk patient populations, underserved areas, and engage with social medicine, thereby leading to the deployment of population health curriculum and the potential for students to understand the extent of the problem.

Overall, continued exposure, practice, and education related to social medicine across medical school will enhance the awareness and knowledge of our students. This will result in better preparation for the zero mile stone residency as meeting one of the ACGME requirements and will eventually lead to the outcome of higher quality physicians in the United States to treat diverse populations. This can and will only be achieved at the medical school level via curriculum reform.
